# Assessing educational needs of sickle cell anemia healthcare providers in sub-Saharan Africa and the Caribbean

**DOI:** 10.3389/fpubh.2025.1693285

**Published:** 2025-12-18

**Authors:** Teresa S. Latham, Russell E. Ware, Lisa Marie Shook

**Affiliations:** 1 Cincinnati Children's Hospital Medical Center, Cincinnati, OH, United States; 2 University of Cincinnati Department of Pediatrics, Cincinnati, OH, United States; 3 Global Health Center, Cincinnati Children’s Hospital Medical Center, Cincinnati, OH, United States

**Keywords:** sickle cell anaemia, global health, capacity building, provider education, hydroxurea

## Abstract

**Objectives:**

Sickle cell anemia (SCA) causes childhood morbidity and mortality in resource-limited settings. Identifying educational needs of healthcare providers allows implementation of targeted programs using evidence-based methods.

**Methods:**

Qualitative data using semi-structured online interviews and surveys were collected from sites across eight countries. Interviews were recorded, transcribed, and coded for thematic analysis.

**Results:**

Eighteen healthcare providers (50% female) from six countries in Africa and the Caribbean were recruited. Four overarching themes emerged: (1) few training opportunities; (2) personal payment for training; (3) busy clinic schedules, so training must occur on personal time; and (4) travel constraints for attending conferences. All participants requested virtual telementoring for continuing education and emphasized an interactive format.

**Conclusion:**

This needs assessment highlights the opportunity to implement telementoring educational programs, such as Project ECHO©, to increase knowledge among multidisciplinary healthcare providers treating children with SCA in resource-limited settings. Providers demonstrated a high level of interest and engagement in education delivered through established North–South partnerships. Telementoring offers an opportunity for capacity building, supporting evidence-based treatment and improving access to knowledgeable providers, ultimately leading to better patient outcomes.

## Background

Sickle cell anemia (SCA) is one of the world’s most common inherited blood disorders and is characterized by a distinct shape change (“sickling”) of red blood cells. This sickling process causes individuals with SCA to experience a wide range of severe and potentially life-threatening clinical complications, including vaso-occlusive painful crises, increased susceptibility to infections, chronic hemolytic anemia, progressive organ damage (including stroke and kidney failure), and early death (1). SCA affects approximately 100,000 individuals in the United States and 12–15 million individuals in low-resource settings across sub-Saharan Africa and the Caribbean (2).

SCA affects individuals throughout their lifespan; however, in the US, under-five mortality has decreased significantly over the past decade due to universal newborn screening and interventions such as penicillin prophylaxis and pneumococcal vaccination. Recent estimates document survival to age 18 years of 93% (3), with a median survival of 42 years for men and 48 years for women (4). However, the burden of SCA remains severe in resource-limited settings, where capacity for diagnosis, prophylaxis, complication screening, and treatment is limited. In these regions, under-5 mortality has been estimated at 6% per 100,000 persons (2).

The WHO provided context for the disease burden in sub-Saharan Africa, noting that in many African countries, approximately 10%–40% of the population carries the sickle-cell gene, resulting in an estimated SCA prevalence of at least 2% (5). The 2021 Global Burden of Disease study reported that total births of babies with SCA increased globally by 13.7% from 2000 to 2021, primarily due to population growth in the Caribbean and in sub-Saharan Africa (2). The incidence of SCA is further projected to increase by 30% by 2050 (6). This increase in disease incidence worldwide, combined with the ongoing high burden of SCA in these settings, constitutes a multi-faceted set of challenges impacting individuals, communities, and health systems, which are collectively burdened by limited infrastructure and lack of resources.

Therapeutic options for SCA are often limited to blood transfusions, which are in limited supply and carry risks to the patient in resource-limited settings. As an alternative to transfusions, hydroxyurea is a once daily oral medication that is safe and effective in conferring clinical benefits and reducing the morbidity of SCA, including pain, acute chest syndrome, and stroke, in addition to reducing mortality in children with SCA (7–9). Hydroxyurea dose-escalation to maximum tolerated dose (MTD) has been shown to be safe and effective in further reducing clinical complications of SCA as compared to fixed dose (10, 11); however, this process is resource-intensive and can take 6–12 months with frequent laboratory monitoring by a trained hematologist or pediatrician.

Knowledge gaps exist among multidisciplinary healthcare providers regarding SCA and disease-modifying therapies, including hydroxyurea and transfusions, which further exacerbate the risk for poor clinical outcomes in children with SCA in resource-limited settings. Studies have found significant challenges in managing SCA in sub-Saharan Africa, specifically the need for healthcare centers to better equip healthcare personnel with adequate training in prevention, management, and treatment of SCA, as well as the need to raise public awareness among communities and within healthcare systems (12). Cross-sectional survey studies of healthcare workers in the Democratic Republic of Congo and Tanzania found inconsistent knowledge of hydroxyurea therapy, pain protocols, and emergency care practices, citing gaps among various levels of providers and identifying resource constraints as major barriers to knowledge acquisition (13, 14). A study in southwestern Nigeria similarly found knowledge among primary healthcare workers to be low to moderate, with significant variation between health facilities and provider levels (15).

Currently, some healthcare providers participating in these clinical trials receive education on the management of patients with SCA and hydroxyurea dosing within the context of clinical research protocols (16). However, this knowledge transfer is limited beyond the scope of the research protocol activities, leaving a majority of healthcare providers lacking the evidence-based knowledge and tools needed to effectively utilize hydroxyurea with SCA patients. Moreover, the knowledge gap continues to widen as newborn screening programs are newly implemented in limited resource areas, and as healthcare providers begin to provide care for children who survive into the teenage years. As the disease progresses, there are new acute and chronic complications that must be managed, as well as challenges associated with growth into adolescence, fertility, and psychosocial support needs, yet there are limited opportunities for targeted, accessible continuing education for healthcare providers.

Currently, there is a gap in the literature about the best strategies to deliver high-quality evidence-based education to healthcare providers about SCA in limited resource settings with established clinical research partnerships. The purpose of this study was to investigate and describe the educational barriers of multidisciplinary healthcare providers treating SCA in resource-limited settings in sub-Saharan Africa and the Caribbean, and to define continuing education needs, including content, prioritization of topics most relevant to their practice, and the dissemination methods that would be most conducive to their uptake.

## Methods

### Design and setting

This mixed-methods study included an online survey or semi-structured interview for multidisciplinary healthcare providers in sub-Saharan Africa and the Caribbean. Participants were recruited from a convenience sampling of healthcare providers from nine clinical sites that have participated in therapeutic trials for children with SCA in collaboration with Cincinnati Children’s Hospital Medical Center. Participants were affiliated with clinical research sites that had existing professional collaborations with two of the study’s co-authors (Latham and Ware), established through prior multi-site research studies. Participants represented four rural clinics in Uganda, Tanzania, and Kenya, and five urban clinics in Angola, the Democratic Republic of Congo, Uganda, and the Dominican Republic, all of which serve as high patient-regional referral facilities for SCA, where many patients often travel long distances for medical care that they cannot obtain close to their homes.

### Ethical considerations

This study received Institutional Review Board (IRB) approval from Cincinnati Children’s Hospital Medical Center (IRB #2023-0655). A waiver of written informed consent was approved. In accordance with international ethical standards and local regulations, IRB approval was not required at the individual institutions from where participants were recruited. This determination was based on the nature of the study, which included interviews with healthcare providers about their own self-reported knowledge and institutional practices, rather than patient data or clinical interventions. All participants provided informed consent verbally prior to the interviews.

To mitigate potential harm in this low-risk qualitative study, several steps were taken, including verbal consent: all participants were provided with detailed information about the study’s purpose, procedures, and their rights, including the right to withdraw at any time without consequence prior to participating in the interview. Confidentiality: Interview data were anonymized, and any identifying information was removed during transcription and thematic analysis. Data were stored securely and accessed only by authorized research personnel. Voluntary participation: Recruitment emphasized the voluntary nature of participation. Cultural sensitivity: Interview guides were reviewed for cultural appropriateness, and local collaborators were consulted to ensure respectful engagement with participants. Minimizing psychological risk: Participants were encouraged to skip any questions they did not wish to answer, and questions were designed to avoid sensitive or distressing topics.

### Data collection

Participants completed a semi-structured recorded interview via Zoom. Interviews were offered in English or Spanish, French, or Portuguese translation options. Trained interviewers performed the semi-structured interviews following an interview guide that included questions across several domains (Supplementary materials) including (1) *demographics* (i.e., participant’s training and practice, scope, and experience and description of their healthcare settings); (2) *training* including informal and formal education about SCA; (3) self-reported *knowledge* about SCA including the evidence-based use of disease-modifying therapy such as hydroxyurea; (4) *educational preferences and needs* including curriculum topics for learning, preferred learning format (i.e., virtual, self-directed, print) and frequency. Interviewers probed about participants’ challenges faced within medical organizations while providing care for children with SCA and opportunities for capacity building, including education. Interviews were transcribed using Zoom transcription tools, cleaned, and then coded for thematic analysis.

Participants who declined virtual interviews were offered the option to complete an online survey created in the REDCap Electronic Data Management system (17, 18). Surveys were made available in English, French, Portuguese, and Spanish and administered based on the self-reported linguistic preferences of the participant. One participant completed a survey in Portuguese, which was translated into English for analysis, and the remainder were completed in English.

### Data analysis

Thematic analysis was conducted using Braun and Clarke’s six-phase framework (19). An inductive approach was used to code interview transcripts, allowing themes to emerge from the data. Two trained researchers independently coded the data manually, and discrepancies were discussed and resolved to reach consensus. No coding software was used. Analysis continued until no new themes emerged, indicating thematic saturation.

## Results

**Figure 1:**
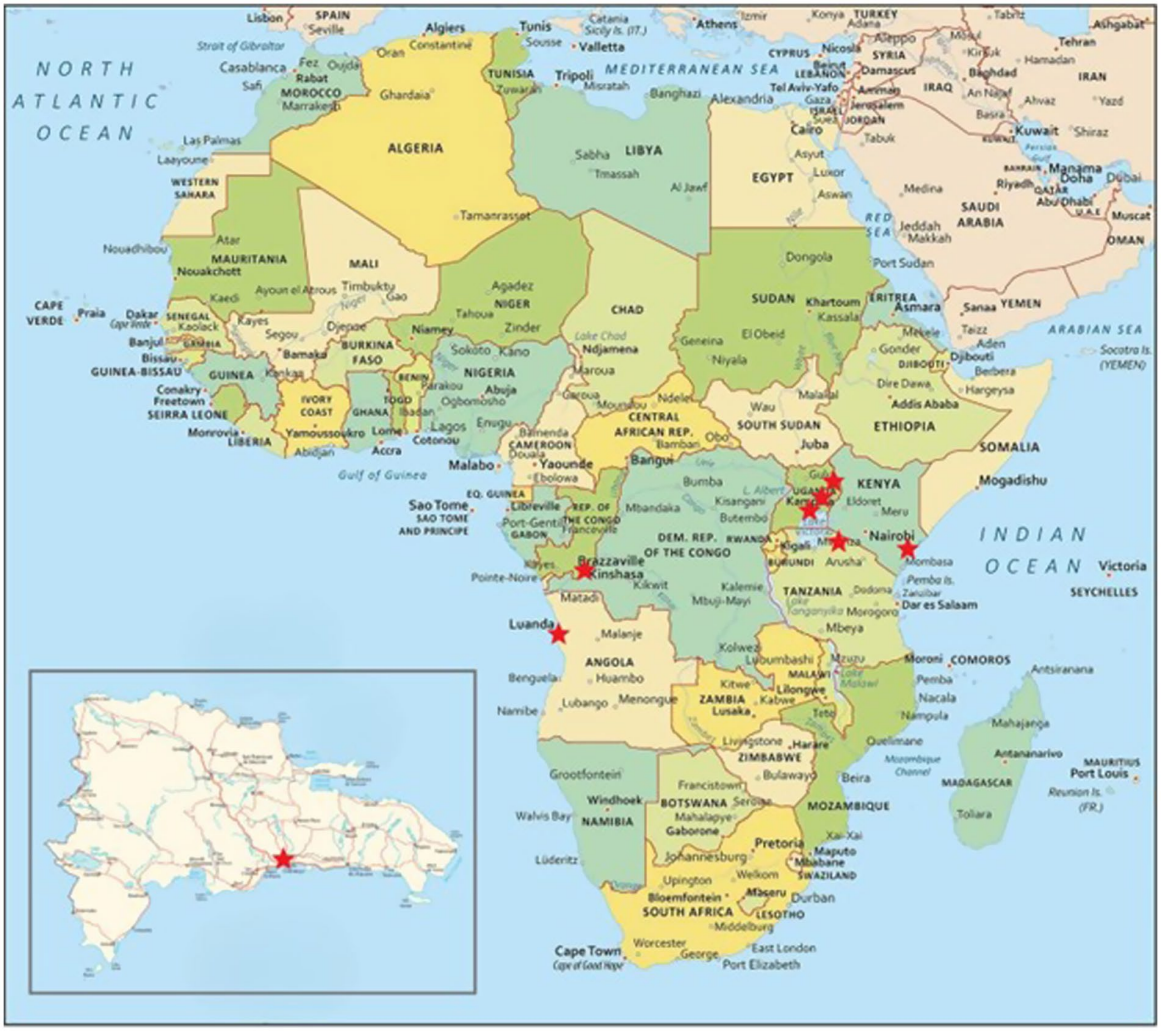
Map of participating sites.

There were 18 participants, representing 5 urban and 7 rural sites from 5 countries, who completed the interviews, plus 6 additional participants who completed surveys, representing 1 urban and 5 sites in 4 countries. Interview and survey participant demographics were from Angola, Democratic Republic of Congo, the Dominican Republic, Kenya, Tanzania, and Uganda, 50% women, and included nine medical doctors, two medical technologists, and one nurse. Interviews lasted an average of 60 min (Figure 1, Table 1).

**Table 1:** Demographics and local setting.

Demographics and local setting	
Respondents	18
Male (*N*, %)	9 (50)
Female (*N*, %)	9 (50)
Age	
Under 30	3 (17)
30–39	11 (61)
40–49	4 (22)
50–59	0 (0)
Role	
Medical doctor	6 (33)
Medical officer	5 (28)
Medical technologist	3 (17)
Nurse	2 (11)
Research coordinator	2 (11)
Setting	
Rural	12 (67)
Urban	6 (33)

Thematic analysis resulted in four overarching themes (Table 2): (1) *clinician-specific experience*, including demographics and local setting descriptions such as clinic information and number of patients seen; (2) *location and context*; (3) *description of resources* currently *available within local settings*, including laboratory capabilities, screening tools such as transcranial Doppler (TCD), psychosocial support, and general educational resources available; and (4) *treatments*, including descriptions of available preventative medications, vaccinations, hydroxyurea, transfusions, and other therapies. Detailed summaries of themes, sub-themes, and educational areas of focus are included in Supplementary materials.

**Table 2:** Themes.

Theme	Description	Codes
Clinician-specific experience	Description of clinician background, demographics, and experience with clinically managing SCA	40
Local setting	Context provided regarding the clinical setting in which participants practice, including setting type, clinic composition (pediatrics vs. adult, facility level), number of patients seen, and provider self-assessment of abilities to provide care for patients with SCA	188
Resources	Description of available resources for diagnosis and treatment of patients with SCA, including lab capabilities, access to screening tools such as TCD, access to supportive care, including psychosocial support, and available resources for continuing education.	244
Treatments	Availability of and provider knowledge about preventative medications such as penicillin prophylaxis, vaccinations, and folic acid and disease-modifying therapies such as hydroxyurea, transfusions, and other treatments for SCA patients.	110

### Clinician-focused experience

The majority of participants reported receiving very little formal education about SCA during their previous medical training, reporting having some basic introductory training, but learning “under fire” by seeing high volumes of SCA patients in their clinics, or case-based learning from other healthcare providers while treating individual patients.

### Clinic capacity and context

Interviews revealed several barriers that impact continuing education among healthcare providers in resource-limited settings, including limited training opportunities on SCA, the need for healthcare providers to pay out-of-pocket for their own training, time constraints with busy clinic schedules that require participation in training during personal time, and travel constraints that limit providers from attending conferences in other geographic locations.

A study participant reported: *“I work in an outpatient clinic. In a week, we might see 150 to almost 160 patients. We come from a very urban area. We’re not that rural. But most of our clients come from the rural areas. So, they really have to travel from afar, so that they can reach this facility. About the setting, I would say, we do not have much, but we are trying to work with whatever we are having most of the time. We have TCDs, but most of it is just basically new to the research purposes. But if you just walk into any outpatient clinic which is basically offering sickle cell anemia diagnostics, or any clinic which helps in diagnosing sickle cell will find out they do not have much. It’s only maybe the dispensing purposes and nothing much.” [SIC].*

Another participant noted: *“We started from nowhere with no folic acid, nothing. We’re just providing them, seeing them, categorizing them and saying, “Okay, this is sickle cell. This might not be sickle cell.” So you do this supportive care, giving patients supportive care. So, then we got folic acid and penicillin, that those are the basic drugs we had for a long time. But now we have hydroxyurea. We started, that is a celebration to me. We started this clinic under tree, now we have a facility where we see them. I would say, that even if we are not there, but we are somewhere.” [SIC].*

Participants reported that technology can be a barrier, such as having to pay for Internet data by the minute out of their own pocket, accessing the Internet on cellphones, which also uses limited data, or unstable Internet connections that can be adversely affected by factors such as rain or other environmental conditions. Furthermore, high Internet traffic during traditional business hours often requires providers to access the Internet very early in the morning or late in the evening, when more bandwidth is available.

### Evidence-based management of SCA

Future training topics of greatest need and interest reported by participants included understanding basic assessments and treatment concepts, common complications of SCA, hydroxyurea dosing, screening tools for complications such as stroke using transcranial Doppler, health education for patients and families, psychosocial support for SCA patients, and the evidence-based management of SCA patients through adolescence and into adulthood, including managing sexual and reproductive health and adherence to treatment. A participant stated: *“…the thing I think what would be most needed here would be lab investigations, understanding complete blood count. If the patients were to easily access investigations such as complete blood counts without waiting very long for results, plus medication, essential sickle cell care medication like hydroxyurea. Sometimes there’s shortages at the facility. Access is inconsistent. For example, I do transcranial Dopplers. but they are not available to the clinic. They’re only available to the research participants. You cannot just look at someone and say, this one will get stroke or not. So, investigations like complete blood count, TCD, even the actual detection of sickle cell with hemoglobin electrophoresis, we need to understand how to use it.” [SIC].*

Another participant described a lack of community knowledge about SCA as further exacerbating challenges experienced by children and their families with the disease: *“For most of them, [with adherence issues], it’s the stigma. At that age most of them are in school, so they are now getting to realize they are taking their own medications every day, if they have any. There’s that aspect of someone will tell you some people are stigmatizing them to other conditions. And then there’s that they have talked about that thing that they are listening to their friends or there’s that stigma kind of people. There are people who tell them is a waste of time. They’re those who the stigma that people with sickle cell do not live long so they’ll kind of tend to maybe lose hope. And just say, even if they take medication, they’ll still not live for long.” [SIC].*

### Future training needs

Participants described the need and opportunity for widespread continuing education about SCA among multidisciplinary healthcare providers beyond clinical research sites. Because of the high volume of individuals living with SCA, particularly in sub-Saharan Africa, there is a need to engage more providers to increase access to care, and to begin to explore developing education for the community at large and other stakeholders to raise awareness about SCA. A participant stated: *“I think the most meaningful will be we need to train more people. We need to train more doctors in sickle cell care because yes, if someone goes to the regional hospital, they will not encounter problems when people have the knowledge and the training on how to properly take care of a sickle cell client and in terms of education to the community that is still needed. We still need to give more education, because even the clients that do come to the hospital, they get discouraged when they go back into society by some people who do not know sickle cell very well, and there’s a lot that has been said even about the medication, and some of them do not adhere to their treatment because of that. So training is needed to the society especially in the periphery, not in the cities, and to the other hospitals that are taking care of these clients.” [SIC].*

While technology barriers require consideration, the majority of participants indicated that they would benefit from virtual telementoring opportunities for continuing education. Several emphasized the importance of an interactive format which encourages dialogue to enhance the didactic lecture format as well as involvement of a range of providers from multiple disciplines and backgrounds as preferable. Participants further emphasized the importance of interacting with other healthcare providers and peers during training to enhance relationship building and the development of a community of practice focused on ongoing learning to improve care for patients with SCA within limited resource settings.

Training about hydroxyurea as a disease-modifying therapy for SCA was emphasized as an essential topic, including hydroxyurea dosing and adherence and the medication’s role in the reduction of pain episodes and other complications. Participants described how hydroxyurea training could be scaled beyond basic knowledge, with a focus on translating knowledge into practice to improve clinical outcomes for patients. They also emphasized that creating a positive impact on families and community members would benefit from providing a basic understanding of the benefits of hydroxyurea and its wide-ranging impact on individuals with SCA and the health systems where they seek care. A participant stated: *“I think, hydroxyurea is more meaningful because it helps with a lot of benefits to patients. I think, dose management first. Education from myself, yes, and education for families.”*

## Discussion

Knowledge of SCA management among healthcare providers at busy, under-resourced clinical sites in sub-Saharan Africa and the Caribbean varies widely. In some cases, increased self-reported provider knowledge about treatment, such as hydroxyurea, results from educational opportunities as a benefit through participating in clinical trials led by sickle cell experts. However, these opportunities are limited and accessible only to a small number of clinical sites within health systems, often due to capacity and resource limitations. The beneficial relationship and collaboration between academic clinical research sponsors and resource-limited clinical sites participating in clinical research studies is well-established, with the benefits of North–South partnerships including mutual respect, trust, and an understanding of cultural context and potential barriers such as technology and infrastructure limitations within these settings well-defined (20, 21).

Self-reported gaps in knowledge present a unique opportunity for the clinical research sponsor site to expand continuing education programs for healthcare providers in these resource-limited settings. The goal is to facilitate the transfer of knowledge and skills from experts and translate this knowledge into practice to improve outcomes for patients. It also provides an opportunity to build communities of practice among healthcare providers, many of whom reported feelings of isolation and not being able to build relationships with providers at other sites. The sites that participated in this needs assessment manage high-volume SCA clinics that may be poorly funded with limited opportunities for continuing education about SCA. However, the participants all indicated a high level of interest and desire for engagement among medical providers of various levels and disciplines working in rural and urban areas, demonstrating that investment in education via North–South partnerships represents an opportunity for capacity building via knowledge transfer in these settings.

To address these knowledge and training gaps, the results of this educational needs assessment suggest the opportunity to implement virtual telementoring continuing educational programs, such as the Project ECHO© framework (22), to increase knowledge and comfort level of healthcare providers treating individuals with SCA in resource-limited settings across sub-Saharan Africa and the Caribbean. This framework started in the United States and has been successfully implemented in Africa for cancer, HIV, and other diseases (23–25). The ability to deliver education via a virtual platform, such as Zoom, WhatsApp, or MS Teams, provides the opportunity for multidisciplinary healthcare providers to participate in learning individually or as a team with hour-long sessions that include a 45-min didactic presentation and a 15-min de-identified case presentation that encourages feedback and recommendations among providers. This continuing education framework would effectively address the barriers described by study participants, such as the inability to travel to conferences because of the lack of time or funding, and isolation within their own hospital or institution.

Addressing the limited knowledge among healthcare providers can create further opportunities to implement educational programs that improve gaps in knowledge among individuals with SCA and ultimately improve clinical outcomes. A study of 1,829 participants with SCA in eastern Uganda reported that while approximately half of the participants knew that SCA was inherited from both parents, a substantial proportion did not know how the disease was transmitted, and many of them believed that SCA is transmitted through blood transfusion. Furthermore, less than 10% were taking hydroxyurea, 20% reported feeling stigmatized, and 80% reported hospital admission for sickle cell-related complications in the past year, all underscoring the need for improved care for sickle cell patients in Uganda (26).

A potential limitation of this study is the small number of participants and sites; however, it is important to recognize that these participants provide healthcare to thousands of individuals with SCA and represent high-volume clinical sites. Moreover, saturation of themes was reached, with consensus among participants about their shared experiences of limited knowledge and educational opportunities despite growing needs across a wide range of topics to improve SCA management. Participants also expressed enthusiasm for the possibility of future continuing education opportunities to improve their knowledge about SCA. Data analysis did not include separating by clinical site or location; however, this may be a relevant consideration in the prioritization of educational programming efforts.

It is also important to note that all participants of this study represent clinical sites in Africa and the Caribbean that have received highly specialized training from two of the study co-authors (Latham and Ware) as part of their participation in multi-site clinical research, indicating that healthcare providers at other clinical sites may have even fewer training opportunities. Inclusion of healthcare workers from settings without specialized training may have provided a broader context for and perspective on educational needs and should be a focus of future research. Several important themes emerged from this study and are likely to be a representative sample that reflects the educational needs and experiences of healthcare providers across other clinical sites in low- to middle-resource settings providing care for individuals with SCA.

## Conclusion

Although SCA is most prevalent in sub-Saharan Africa, there are still significant self-reported generalized knowledge gaps and evidence-based management gaps, along with scarce opportunities for continuing education. These knowledge gaps present a unique opportunity to design tailored virtual telementoring educational programs for healthcare providers in limited resource settings. Project ECHO© telementoring is designed to reach multiple healthcare providers across geographical locations to provide evidence-based education, as well as to create a community of practice among healthcare providers who may not otherwise have the opportunity to interact with other healthcare providers caring for individuals with SCA due to limited resources. Moreover, there is the opportunity to capitalize on existing relationships and collaborations between academic medical centers in the United States with clinical research sites in sub-Saharan Africa and the Caribbean to spread the diffusion of knowledge and implement this learning network. Knowledge transfer through a telementoring framework using the established ECHO model has the potential to build capacity within resource-limited settings through a feasible, sustainable platform that can be further spread within healthcare systems and communities.

Designing a robust educational curriculum based on interview responses from multidisciplinary providers ensures that the training provided is appropriate within the local contexts and thus likely to have significant uptake and benefits to providers and ultimately the patients for whom they provide care. SCA represents an underserved population whose outcomes may be improved by addressing the multiple capacity gaps and healthcare system challenges that exist in resource-limited settings. Improved educational opportunities on topics most relevant to providers within these settings have the potential to improve outcomes and build capacity within health systems where the need is greatest.

## Data Availability

The raw data supporting the conclusions of this article will be made available by the authors, without undue reservation.
