# Supplementary material for: Assessing educational needs of sickle cell anemia healthcare providers in sub-Saharan Africa and the Caribbean
Source: Front Public Health. 2025 Dec 18;13:1693285. doi: 10.3389/fpubh.2025.1693285 (PMC12756480; doi:10.3389/fpubh.2025.1693285)
Supplement: Supplementary file 1 [file Data_Sheet_1.docx]

**Supplemental Table 1, Detailed Data Table of Themes and Subthemes**

| **Theme** | **Subtheme** | **Coded Statements** | **Representation Within Theme (%)** | **Representation Overall (%)** |
| --- | --- | --- | --- | --- |
| **Clinician** |  | **40** |  |  |
|  | Demographics | 15 | 37.5 | 2.6 |
|  | Experience and Background | 15 | 37.5 | 2.6 |
|  | Competency | 10 | 25.0 | 1.7 |
| **Local Setting** |  | **180** |  |  |
|  | Cost of Care | 28 | 15.6 | 4.9 |
|  | Local Setting | 21 | 11.7 | 3.6 |
|  | Health literacy of patients | 18 | 10.0 | 3.1 |
|  | Quality/Limitations in Care | 15 | 8.3 | 2.6 |
|  | Facility | 13 | 7.2 | 2.3 |
|  | Patient Volume | 13 | 7.2 | 2.3 |
|  | Government, Laws | 12 | 6.7 | 2.1 |
|  | Burden of SCA on the healthcare system | 11 | 6.1 | 1.9 |
|  | Community | 10 | 5.6 | 1.7 |
|  | Burden of SCA for the patient | 9 | 5.0 | 1.6 |
|  | Patient Demographics | 9 | 5.0 | 1.6 |
|  | stigma | 5 | 2.8 | 0.9 |
|  | Burden of SCA on the community | 4 | 2.2 | 0.7 |
|  | Equity | 4 | 2.2 | 0.7 |
|  | Prevalence | 4 | 2.2 | 0.7 |
|  | Other patient needs | 2 | 1.1 | 0.3 |
|  | Burden of SCA on the community | 1 | 0.6 | 0.2 |
|  | Trait Screening | 1 | 0.6 | 0.2 |
| **Resources** |  | **208** |  |  |
|  | Ongoing Training | 132 | 63.5 | 22.9 |
|  | TCD | 17 | 8.2 | 3.0 |
|  | Supplies | 16 | 7.7 | 2.8 |
|  | Impact of Clinical Trials | 15 | 7.2 | 2.6 |
|  | Travel for Care | 8 | 3.8 | 1.4 |
|  | Staffing | 8 | 3.8 | 1.4 |
|  | Needs | 4 | 1.9 | 0.7 |
|  | Newborn Screening | 4 | 1.9 | 0.7 |
|  | Quality/Limitations in Care | 3 | 1.4 | 0.5 |
|  | Funding | 1 | 0.5 | 0.2 |
| **Treatment** |  | **148** |  |  |
|  | Hydroxyurea | 53 | 35.8 | 9.2 |
|  | Technology | 40 | 27.0 | 6.9 |
|  | Other treatments | 30 | 20.3 | 5.2 |
|  | Adherence | 15 | 10.1 | 2.6 |
|  | Trait Screening | 4 | 2.7 | 0.7 |
|  | Antibiotics | 3 | 2.0 | 0.5 |
|  | Cost of Care | 1 | 0.7 | 0.2 |
|  | Immunizations | 1 | 0.7 | 0.2 |
|  | Other | 1 | 0.7 | 0.2 |

**Supplemental Table 2. Self-Reported Healthcare Provider Continuing Education Needs**

| **Training Needs** | **Representation within Sub-Theme (%)** |
| --- | --- |
| Format | 22.0 |
| Immunizations, antibiotics, folic acid | 12.1 |
| Pain | 9.8 |
| Adherence | 9.1 |
| complications | 9.1 |
| Hydroxyurea | 9.1 |
| TCD, stroke | 7.6 |
| Training new providers | 4.5 |
| Other topics | 3.0 |
| Reproductive health, sexual health, mental health | 3.0 |
| Timing | 3.0 |
| Nutrition | 2.3 |
| Technology | 2.3 |
| Stigma | 1.5 |
| Public outreach | 0.8 |
| Misc topics | 0.8 |
